# The Therapeutic Effects of a PEDF-Derived Short Peptide on Murine Experimental Dry Eye Involves Suppression of MMP-9 and Inflammation

**DOI:** 10.1167/tvst.11.10.12

**Published:** 2022-10-06

**Authors:** Tsung-Chuan Ho, Nai-Wen Fan, Shu-I Yeh, Show-Li Chen, Yeou-Ping Tsao

**Affiliations:** 1 Department of Medical Research, Mackay Memorial Hospital, New Taipei City, Taiwan; 2 Department of Ophthalmology, Taipei Veterans General Hospital, Taipei, Taiwan; 3 Faculty of Medicine, National Yang Ming Chiao Tung University, Hsinchu, Taiwan; 4 Department of Medicine, Mackay Medical College, New Taipei City, Taiwan; 5 Department of Ophthalmology, Mackay Memorial Hospital, Taipei, Taiwan; 6 Graduate Institute of Microbiology, College of Medicine, National Taiwan University, Taipei, Taiwan

**Keywords:** dry eye disease, PEDF, receptor, peptide, MMP-9, inflammation

## Abstract

**Purpose:**

To evaluate the efficacy of a pigment epithelium-derived factor (PEDF)-derived short peptide 29-mer, on the treatment and prevention of experimental dry eye (EDE).

**Methods:**

C57BL/6 mice were housed in a low humidity controlled environment chamber for 14 days to induce EDE. The 29-mer was administered topically to their eyes, for treatment or dosing, from the point of housing in the controlled environment chamber. The efficacy of the 29-mer on EDE was evaluated in terms of corneal epithelial integrity, tear secretion, and the density of conjunctival goblet cells. PEDF and inflammatory factors, including tumor necrosis factor-α, IL-1β, IL-6, monocyte chemotactic protein (MCP)-1, matrix metalloproteinase-9, and macrophage infiltration, were examined by real-time polymerase chain reaction, Western blotting, and immunostaining. The involvement of the PEDF receptor/PNPLA2 on the 29-mer effects was evaluated by a specific inhibitor, atglistatin. Rabbit corneal epithelial cells were exposed to hyperosmotic medium to induce inflammatory responses.

**Results:**

The levels of PEDF protein increased in the corneal epithelium of EDE, compared with the nonstressed mice. The 29-mer showed a therapeutic effect on EDE and prevented the development of EDE, accompanied by amelioration of the inflammatory factors. The 29-mer effects of inflammatory relief were dramatically reversed by atglistatin. The 29-mer also suppressed the expression of matrix metalloproteinase-9 and proinflammatory cytokines in rabbit corneal epithelial cells induced by hyperosmolarity.

**Conclusions:**

Through this animal study, we provide a proof of concept of the anti-inflammatory domain of PEDF having potential to treat dry eye disease.

**Translational Relevance:**

This study shows the 29-mer has novel potential as an ophthalmic drop treatment for dry eye disease.

## Introduction

Dry eye disease (DED) is a common ocular disorder, affecting the patient's quality of life with various symptoms, including gritting of the eyes, transient blurred vision, persistent eye pain, and even loss of vision.^1^ DED is often associated with decreased tear secretion and/or increased tear evaporation that cause loss of tear film homeostasis, elevation of tear osmolarity, and disruption of corneal barrier function at the ocular surface.^2^ The hyperosmotic dry eye milieu is known to be accompanied by inflammatory changes.^2^ For example, various inflammatory mediators, including matrix metalloproteinases (MMPs), tumor necrosis factor-α (TNF-α), interleukin (IL)-1β, and IL-6 are induced by the hyperosmolarity.^3^^–^^5^ Among these, the levels of MMP-9 in the dry eye have been shown to correlate strongly with the severity of DED and may be decreased by adequate treatment.^6^^,^^7^

The anti-inflammatory functions of recombinant pigment epithelium-derived factor (rPEDF) have been demonstrated using murine models of experimental dry eye (EDE).^8^^,^^9^ The therapeutic actions of rPEDF on EDE are reportedly associated with suppression of the expression of proinflammatory cytokines, impeding the maturation of dendritic cells and blocking the amplification of T helper 17 cells in the draining lymph nodes of EDE mice, but its effect on MMP-9 expression remains to be elucidated.^8^^,^^9^ In addition, the expression of endogenous PEDF is reportedly increased in the corneal epithelium in EDE and in the tears of patients with dry eye.^8^^,^^9^ Our recent study found that a PEDF-derived peptide, the 29-mer (residues Ser93–Thr121; SLGAEQRTESIIHRALYYDLISSPDIHGT), was able to induce the proliferation of acinar basal cell in the atrophied meibomian glands of aged mice, thereby enabling recovery of the stability of the tear film lipid layer, potentially enabling DED therapy.^10^

PEDF is known to exert multiple functions by interacting with various cell surface receptors (PEDF-R). For example, the 37/67-kDa laminin receptor is involved in mediating the angioinhibitory effect of PEDF.^11^ The association of PEDF with the LRP6 receptor causes blockage of Wnt-mediated liver fibrosis.^12^ Of note, one of the PEDF-R, patatin-like phospholipase domain containing 2 (PNPLA2), has a calcium-independent phospholipase activity induced by PEDF.^13^^,^^14^ PNPLA2 has been shown to be expressed in the mouse corneal epithelium.^15^ In addition, PEDF is beneficial for corneal epithelial wound healing via PNPLA2-mediated intracellular signaling, although the therapeutic effect of PEDF is blocked by a specific PNPLA2 inhibitor, atglistatin.^15^ Furthermore, the PNPLA2-deficient mouse has been shown to suffer from hepatic inflammation and the development of atherosclerotic lesions, highlighting an anti-inflammatory role of PNPLA2 stimulated by PEDF.^16^^,^^17^ However, the role of PEDF-R in the therapeutic effect of PEDF on EDE has not been explored.

The core domain responsible for the anti-inflammatory actions of PEDF on EDE remains to be delineated. This effort is critical for understanding the action of PEDF on DED, because small synthetic peptides containing the active core domain may exclude potential interference from other domains of PEDF in vivo and increase the ability of the drug to diffuse into the dry eye boundary.^18^ In this study, we investigated whether the 29-mer has a potential role in the treatment of EDE. Rabbit corneal epithelial cell (RCECs) were also used to investigate the mechanisms linking the 29-mer with hyperosmotic stress-induced inflammatory responses.

## Methods

### Materials

Periodic acid-Schiff (PAS) kits and all chemicals were from Sigma-Aldrich (St. Louis, MO). Antibodies against PEDF, MMP-9, F4/80, and occludin were from Abcam (Cambridge, MA). PEDFR/PNPLA2 Antibody (Cat# AF5365) was from R&D systems (Minneapolis MN). Phospho-SAPK/JNK (Thr183/Tyr185) and SAPK/JNK were from Cell Signaling Technology (Danvers, MA). SB203580 and SP600125 were from Selleckchem (Houston, TX). The 29-mer peptide was modified for stability by acetylation at the NH_2_ terminus and amidation at the COOH terminus and synthesized at GenScript (>95% purity; Piscataway, NJ).

### Animal Studies

Standard laboratory chow and tap water were available ad libitum. Experimental procedures were approved by the Mackay Memorial Hospital Review Board (project code: MMH-A-S-107-52; New Taipei City, Taiwan). Animals in ophthalmic research were treated in compliance with the ARVO Statement and the ARRIVE Guidelines. Eight-week-old female C57BL/6 mice were supplied by the BioLASCO (Taiwan) Mice were grouped randomly (*n* = 3–8 per group) and data were assessed from both eyes per animal. Mice were anaesthetized by an intraperitoneal injection of a mixture of ketamine (20 mg/kg body weight) and xylazine (10 mg/kg). All animals were euthanized by CO_2_ inhalation before dissection.

### Induction of EDE and Eye Drop Treatment

EDE was induced as described in a previous study.^19^ Briefly, controlled environment chamber (CEC) conditions were controlled to provide a relative humidity of less than 25%, airflow of 15 L/min, and temperature of 24°C to 25°C, continuously. Age- and sex-matched nonstressed (NS) mice were housed in normal ambient conditions. Balanced salt solution (BSS, Alcon Laboratories, Fort Worth, TX) was used as the vehicle for eye drops. We administered 10 µL of 100 µM peptide or vehicle topically to the eyes three times daily.

### Corneal Fluorescein Staining of EDE

Sodium fluorescein (Alcon Laboratories) was instilled via a micropipette into the inferior–lateral conjunctival sac (0.6 µL of 0.5% fluorescein dissolved in 4.4 µL of phosphate-buffered saline, per eye). After staining for 15 seconds, mouse eyes were washed once with phosphate-bufferd saline and then examined using a slit-lamp microscope with cobalt blue light (Topcon SL-7F, Tokyo, Japan). Punctate staining was evaluated in a masked fashion, giving a score of 0 to 3 to each cornea: a score of 0 for no punctate staining, a score of 1 when less than one-third of the cornea was stained, a score of 2 when two-thirds or less was stained, and a score of 3 when more than two-thirds were stained.

### Measurement of Tear Production

Tear production was measured with phenol red–impregnated cotton threads (Zone-Quick; Oasis, Glendora, CA). The validity of this test in mice was established previously.^20^ The threads were held with jewelers’ forceps and placed in the lateral canthus for 60 seconds. The tear levels were expressed as millimeters of thread, wetted and turned red by the tears.

### PAS Staining of Goblet Cells

After the animals were euthanized, eyes and ocular adnexa were excised surgically, fixed in 4% paraformaldehyde, embedded in paraffin, and cut into 8-µm sections. To measure goblet cells in the superior and inferior conjunctiva, the sections were stained with PAS reagent according to manufacturer's protocol. Images were captured using a Nikon Eclipse 80i microscope (Nikon Corporation, Tokyo, Japan) equipped with a Leica DC 500 camera.

### Immunofluorescence

Deparaffinized tissue sections or 4% paraformaldehyde-fixed RCECs were blocked with 10% goat serum and 5% bovine serum albumin in phosphate-buffered saline/Tween for 20 minutes. Staining was performed using primary antibodies diluted to 1:100 in phosphate-buffered saline/Tween at 37°C for 3 hours. The sections were viewed with an epifluorescence microscope (Zeiss, Oberkochen, Germany) equipped with a charge-coupled device camera.

### Cell Culture and Treatment

RCECs were isolated from 6-month-old New Zealand white rabbits and continuously cultivated for 14 days in Dulbecco's modified Eagle's medium/F-12 basal medium to achieve corneal-liked epithelial cell differentiation, as previously described.^21^ To induce hyperosmotic stress, the cells were incubated overnight in hypertonic media (463 mOsm) by the addition of 90 mM NaCl. Cells cultured in the basal medium (309 mOsm) were used as negative controls. Cells were also pretreated with 10 µM of 29-mer for 6 hours before treatment with NaCl.

### Quantitative Real-Time Polymerase Chain Reaction

Total RNA extraction, synthesis of cDNA, and quantitative polymerase chain reaction (qPCR) were performed as previously described.^22^ Primers used in the experiment are listed in the Table. All determinations were made in triplicate. The threshold cycles measured with interest genes were normalized to the geometric mean of two reference genes, glyceraldehyde-3-phosphate dehydrogenase (*GAPDH*) and hypoxanthine guanine phosphoribosyl transferase (*Hprt*), according to the comparative threshold cycles method.^23^^,^^24^

**Table.** Primers Used for Real-Time qPCR

Target Gene	Primer (Sense)	Primer (Antisense)	Accession No.
moTNF‐α	5′‐ CCAAATGGCCTCCCTCTCAT	5′‐ CACTTGGTGGTTTGCTACGA	NM_013693.3
moIL‐1β	5′‐ GGCTCATCTGGGATCCTCTC	5′‐ GTTTGGAAGCAGCCCTTCAT	NM_008361.4
moIL‐6	5′‐ GGAGCCCACCAAGAACGATA	5′‐ ACCAGCATCAGTCCCAAGAA	NM_031168.2
mMCP-1	5′- CTCGGACTGTGATGCCTTAAT	5′- TGGATCCACACCTTGCATTTA	NM_011333
moMMP-9	5′- GCAGAGGCATACTTGTACCG	5′- ATGGCCTTTAGTGTCTGGCT	NM_013599.4
mGAPDH	5′‐ AACGGATTTGGCCGTATTGG	5′‐ CATTCTCGGCCTTGACTGTG	NM_001289726.1
mHPRT	5′‐ GCTTACCTCACTGCTTTCC	5′‐ TTCATCATCGCTAATCACGAC	NM_013556.2
RbTNF‐α	5′- CCCTTAGGGAGCAGAGGTTC	5′- CCTGTGCCTCCCTTCACTTA	NM_001082263.1
RbIL‐1β	5′- CCTGTTCTTTGAGGCCGATG	5′- GCCGGAAGCTCTTGTTGTAG	NM_001082201.1
RbIL‐6	5′- GCAGAACCATCGAGAGCATC	5′- CAGATTGACTTCCGCCAGTG	DQ680161.1
RbMCP-1	5′- CTCTCACCCTCCAGCATGAA	5′- CAAGCACATGGGAGCTGAAG	NM_001082294.1
RbMMP-9	5′- TGCGAGTTTCCGTTCATCTT	5′- GTAGAGCTTGTCCTTGTCGTAG	NM_001082203
RbGAPDH	5′- AGGTCATCCACGACCACTTC	5′- GTGAGTTTCCCGTTCAGCTC	NM_001082253.1
RbHPRT	5′- GTTCTTTGCTGACCTGCTG	5′- TCCACCGATTACTTTTATGTCC	EF062857.1

### Western Blot Analysis

Cell lysis and sodium dodecyl sulfate polyacrylamide gel electrophoresis were performed as described previously.^22^ The band intensities in immunoblots were measured with a Model GS-700 imaging densitometer (Bio-Rad Laboratories, Hercules, CA) and analyzed using Labworks 4.0 software.

### Gelatin Zymography

To detect MMP-9 activity, 10 µL of culture medium was used for perform gelatin zymography, as previously described.^4^

### Statistics

The results are expressed as mean ± standard deviation. Comparisons of controls and the treatment groups were performed using the Mann-Whitney *U* test. A *P* value of less than 0.05 was considered significant.

## Results

### Topical 29-mer Treatment Benefits the Healing of Ocular Surface Damage and Recovery of Tear Secretion in EDE Mice

**Figure 1.**
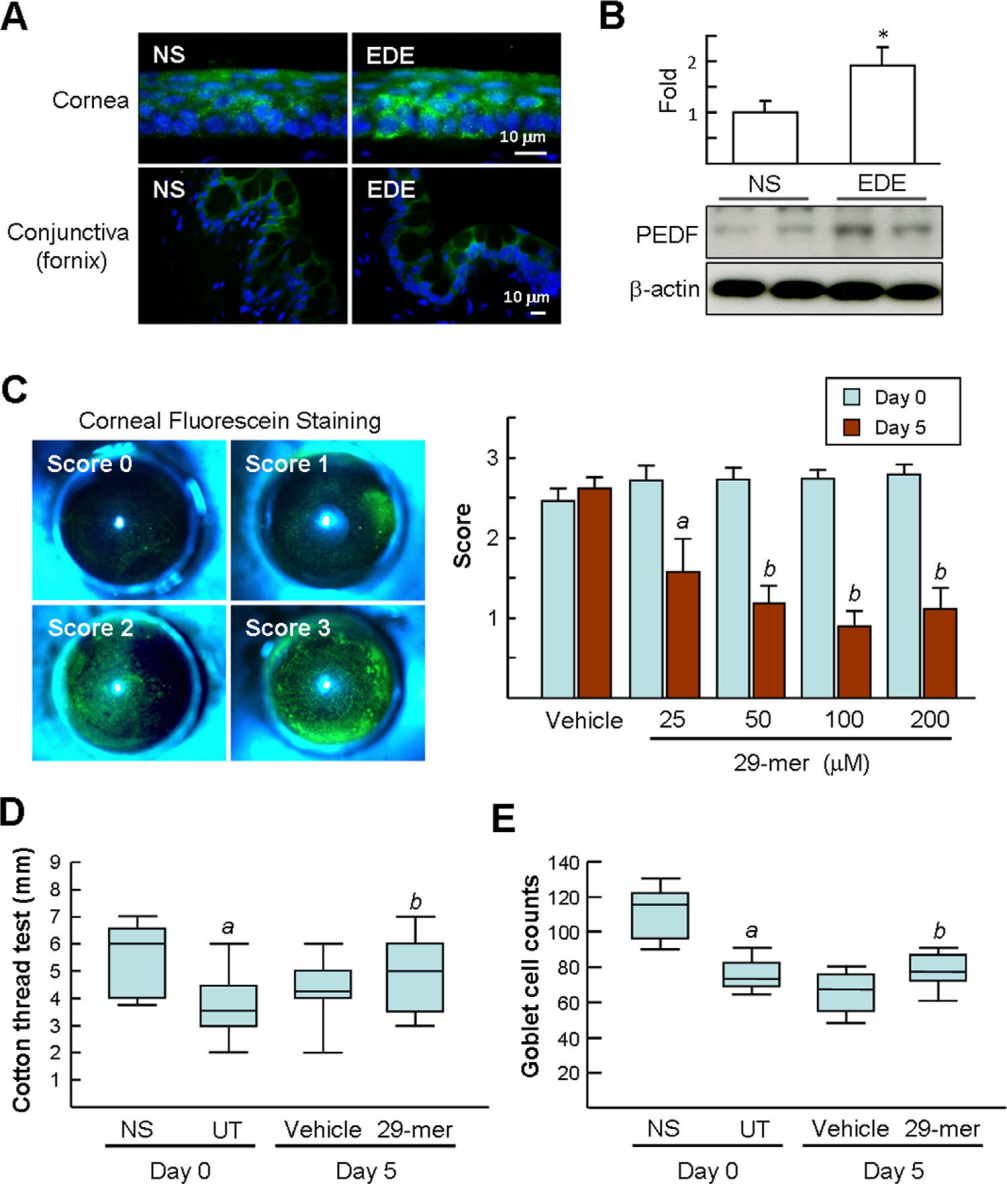
Amelioration of murine EDE by topical treatment with the 29-mer. C57BL/6 mice were housed in a low humidity CEC for 14 days to induce EDE (day 0) and then topically administered various doses of 29-mer or vehicle eye drops for an additional 5 days (day 5). **(A)** Representative immunofluorescence staining of PEDF after 14 days of EDE induction (*n* = 3 per group). Nuclei were stained by Hoechst 33258 (*blue*). **(B)** Representative Western blots from three independent experiments and densitometric analyses of PEDF in tissues from ocular surface epithelia. **(C)** Representative images of fluorescein staining used for scoring the corneal epithelial damage and statistical analysis of the mean scores after treatment with different doses of the 29-mer. Eight animals per condition were analyzed. *^a^P* < 0.03 versus day 0. *^b^P* < 0.00001 versus day 0. **(D)** Analysis of the amount of tear production by phenol red–impregnated cotton threads and statistically depicted by a box and whisker plot. Data show median and interquartile ranges of individual mice (*n* = 8 per group). UT, untreated (mice housed under CEC for 14 days). *^a^P* = 6.36E-06 versus NS. *^b^P* = 0.01 versus vehicle group. **(E)** The number of goblet cells in conjunctiva observed by PAS staining (*n* = 8 per group). *^a^P* = 2.03E-14 versus NS. *^b^P* = 0.002 versus vehicle group.

Mice were housed in a low humidity CEC for 14 days to induce EDE. Immunofluorescence staining of PEDF showed its levels increased in all layers of the corneal epithelium of EDE, especially in the basal layer cells, compared with NS mice (Fig. 1A). The distribution of PEDF at the conjunctiva was similar in the two groups, but with diverse intensities compared with the corneas. Western blotting of the ocular surface tissues confirmed the levels of PEDF in EDE were 1.9 ± 0.4-fold higher than in NS mice (Fig. 1B). The results also imply that the increase of endogenous PEDF in the ocular surface is not sufficient to block the development of EDE.

We investigated the therapeutic effect of the 29-mer on EDE by topical treatment for another 5 days. Subsequently, the integrity of the corneal epithelium was estimated using fluorescein staining. The 29-mer reduced the corneal fluorescein score, compared with the vehicle group, and in a dose-dependent manner (25–200 µM; 0.9–1.6 vs. 2.5–2.8) (Fig. 1C). Decreased tear secretion was also evident in mice housed in the CEC for 14 days (day 0) compared with NS mice (3.6 mm vs. 6.0 mm) (Fig. 1D). The impairment of tear secretion was reversed by treatment with 100 µM of 29-mer, but not by vehicle treatment (*P* < 0.01). EDE mice at day 0 also had reduced numbers of goblet cells in the conjunctiva, compared with NS mice (median number of 74 vs. 115) (Fig. 1E). After further treatment for 5 days, goblet cells in vehicle-treated eyes progressively reduced in number (median, 68). In contrast, the 29-mer significantly blocked this loss (median, 75; *P* < 0.002). Taken together, the 29-mer ameliorates the ocular surface defects in EDE.

### Topical 29-mer Treatment Prevents the Pathogenesis of EDE

**Figure 2.**
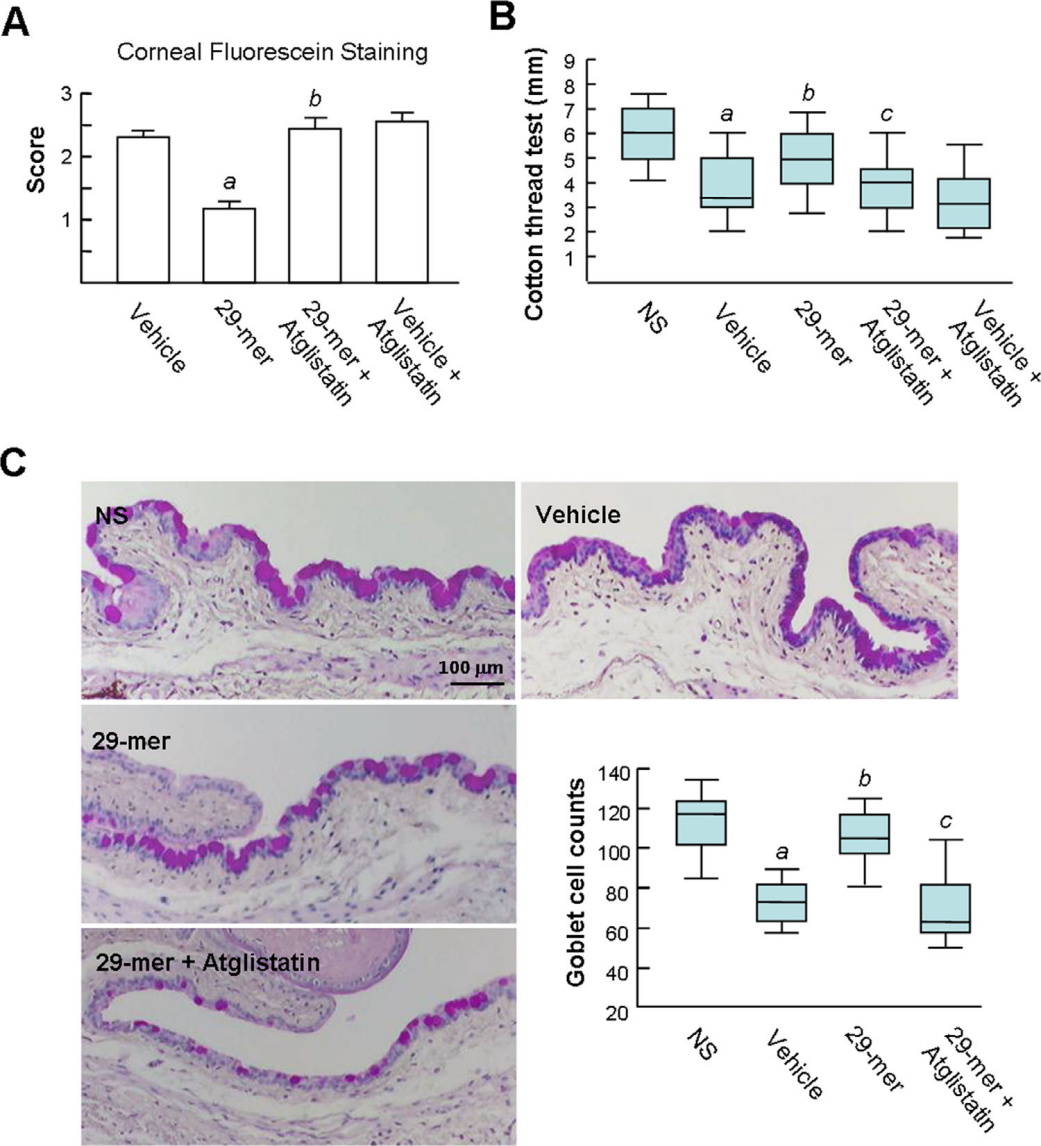
The 29-mer prevents the development of EDE. Treatment with 100 µM of 29-mer was started at the beginning of mice housed in CEC for 14 days. Treatment with 50 µM atglistatin eye drops was performed before 29-mer treatment for 30 minutes. **(A)** The mean score of corneal fluorescein staining at day 14. Eight animals per condition were analyzed. *^a^P* = 7.46E-09 versus the vehicle group. *^b^P* = 6.82E-07 versus the 29-mer group. **(B)** Tear production assayed by cotton threads test (*n* = 8 per group). *^a^P* = 1.03E-07 versus NS. *^b^P* = 0.008 versus vehicle. *^c^P* = 0.01 versus 29-mer. **(C)** Representative images of goblet cells in conjunctiva observed by PAS staining and statistical analysis of numbers of goblet cells depicted by box plots (*n* = 8 per group). *^a^P =* 8.11E-13 versus NS. *^b^P* = 1.88E-11 versus vehicle. *^c^P* = 5.22E-11 versus 29-mer.

Next, 29-mer dosing was started at the point of housing the mice in the CEC for 14 days. First, an analysis of corneal fluorescein–stained EDE demonstrated that the impairment of the corneal epithelial integrity was decreased by 29-mer treatment, compared with vehicle-treated eyes (1.2 ± 0.13 vs. 2.3 ± 0.11) (Fig. 2A). The 29-mer also significantly prevented the decrease of tear production, compared with the vehicle group (*P* = 0.008) (Fig. 2B). In addition, PAS staining of goblet cells showed a continuous homogeneous pattern at the conjunctival fornix in the NS eye, but a dramatic decrease in vehicle-treated eyes (median number, 117 vs. 74) (Fig. 2C). The 29-mer prevented the loss of goblet cells (median number, 104). Of note, all of the protective effects of the 29-mer were almost completely blocked by atglistatin, implying a critical involvement of PEDF-R/PNPLA2 in the protective signaling induced by the 29-mer.

### The 29-mer Suppresses the Induction of MMP-9 at the Ocular Surface by Desiccation, in a PEDF-R–Dependent Manner

**Figure 3.**
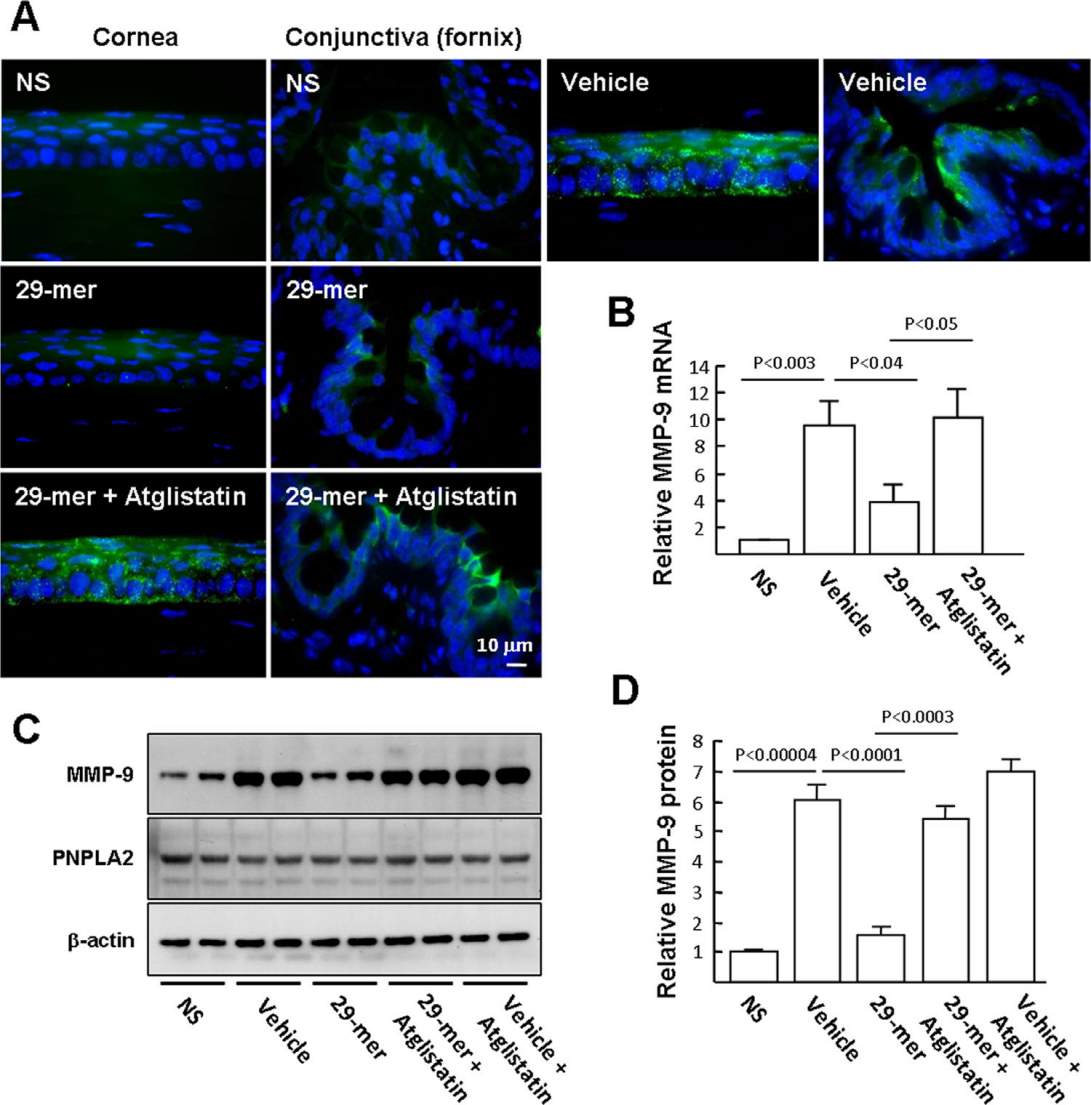
Effect of the 29-mer on the expression of MMP-9 in EDE. The schedule of induction of EDE is described in Fig. 2. **(A)** Representative immunofluorescence images showing MMP-9 in corneal and conjunctival epithelia after 14 days of EDE induction (*n* = 6 per group). **(B)** qPCR evaluates the levels of MMP-9 in the ocular surface epithelia (*n* = 6 per group). **(C** and **D)** Representative Western blots and densitometric analyses of MMP-9 and PEDF-R of ocular surface epithelia are shown from three independent experiments.

The expression of MMP-9 is induced in EDE, triggering a key challenge to the corneal epithelial barrier.^4^^,^^25^ Immunofluorescence staining of MMP-9 showed that vehicle-treated eyes had a strong fluorescence density in the corneal epithelium and some areas of the conjunctival fornix, compared with weak staining patterns in the NS group and 29-mer–treated eyes (Fig. 3A). The expression of MMP-9 in the ocular surface epithelia, assayed by qPCR and Western blotting, showed that gene and protein expression increased in the vehicle group by more than 9.6-fold and 6.1-fold, respectively, compared with the NS group (Figs. 3B–D), whereas this induction was significantly suppressed by 29-mer treatment. Western blots also revealed that the levels of PNPLA2 in the ocular surface of EDE were similar to the NS control. Meanwhile, the inhibitory effects of 29-mer on MMP-9 expression were reversed by atglistatin, suggesting a critical requirement of PNPLA2 for MMP-9 down-regulation.

### The 29-mer Suppresses the Hyperosmotic Stress-Induced Expression of MMP-9 by RCECs

**Figure 4.**
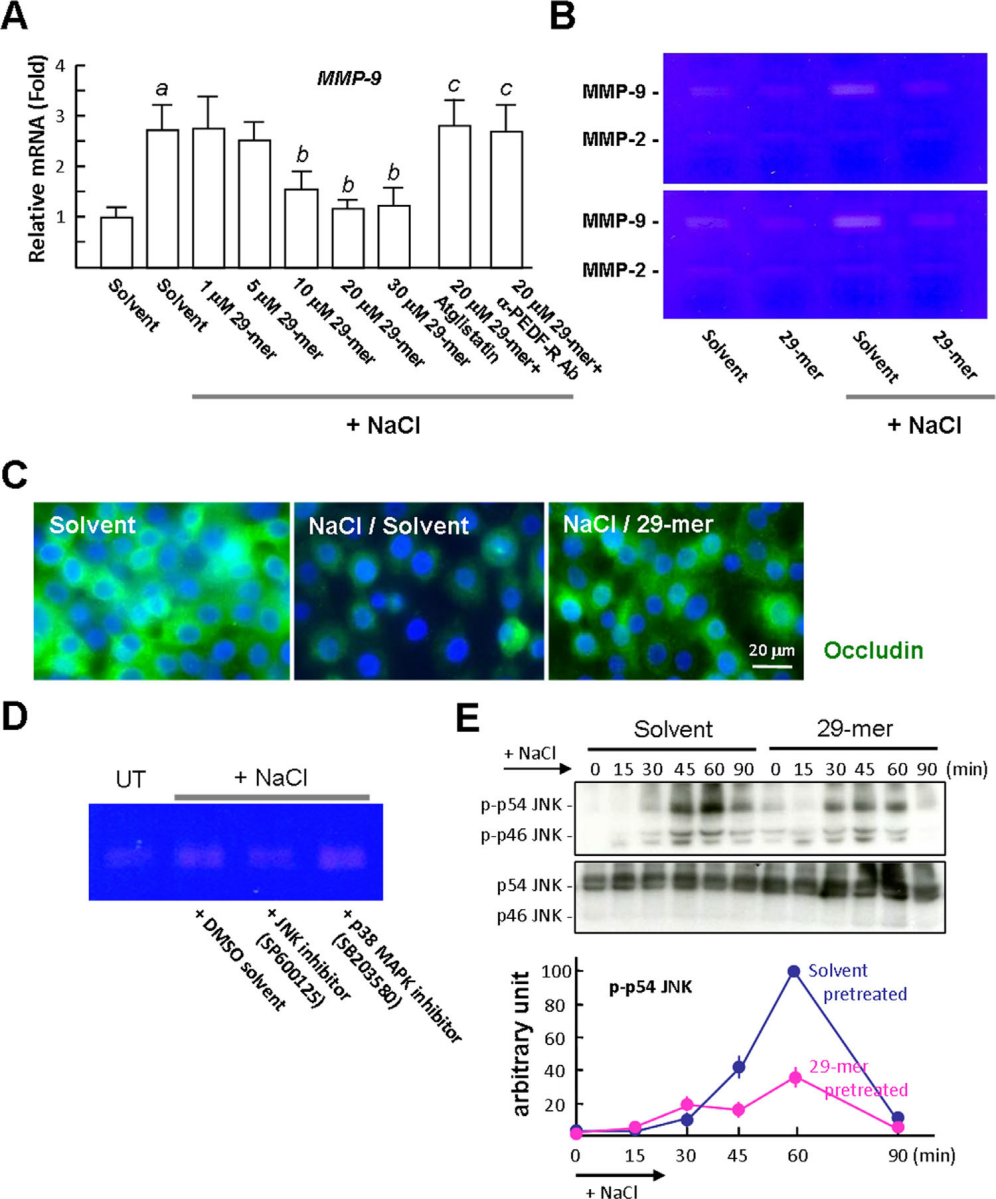
The 29-mer down-regulates the expression of MMP-9 in RCECs exposed to hyperosmotic medium. RCECs were treated with the 29-mer for 6 hours or pretreated with 10 µM atglistatin or a neutralizing Ab (5 µg/mL) against PEDF-R for 30 minutes before 29-mer treatment, and then switched to hyperosmotic medium by adding 90 mM NaCl. (**A**) After NaCl treatment for 6 hours, MMP-9 mRNA levels were evaluated by qPCR. Average levels of MMP-9 from three separate experiments are shown. *^a^P* < 0.02 versus Solvent. *^b^P* < 0.05 versus solvent + NaCl. *^c^P* < 0.03 versus 29-mer + NaCl. **(B)** After NaCl treatment for 24 hours, levels of MMP-9 in the cultured media of RCECs were evaluated by gelatin zymography. **(C)** Representative immunofluorescence images of occludin (*green*) in RCECs after NaCl treatment for 24 hours. **(D)** Gelatin zymography. Cells were pretreated with 10 µM inhibitors for 30 minutes before 29-mer treatment. **(E)** Representative Western blots and densitometric analyses of JNK phosphorylation and total JNK. Data are summarized from three separate experiments.

The expression and production of MMP-9 by cultured CECs is reportedly induced by the addition of NaCl to trigger hyperosmotic stress.^4^ NaCl treatment caused a 2.8-fold increase in *MMP-9* gene expression, compared with the solvent control (Fig. 4A). Pretreatment of the cells with 10 to 30 µM of 29-mer significantly decreased the NaCl-stimulated *MMP-9* gene expression to near basal levels. The effect of the 29-mer was dramatically reversed by atglistatin and PEDF-R/PNPLA2–blocking antibody. Gelatin zymography revealed that the levels of 92-kDa MMP-9 in the culture media were increased by NaCl treatment, compared with solvent treatment alone (approximately 4-fold) (Fig. 4B). The 29-mer treatment caused the production of MMP-9 to return to basal levels. In contrast, the production of 72-kDa MMP-2 was increased less by hyperosmotic stimulation, as described in a previous report.^4^ Immunofluorescence staining of occludin, a tight junction protein, showed that the signal was more intense in 29-mer/NaCl–treated cells than solvent/NaCl–treated cells (Fig. 4C), correlating positively with the decrease in MMP-9 production in 29-mer/NaCl–treated cells.

The increase of MMP-9 in the culture media was completely blocked by c-jun N-terminal kinases (JNK) inhibitor, but was not affected by p38 MAPK inhibitor (Fig. 4D). Western blotting revealed that JNK phosphorylation was induced after hyperosmotic stimulation for 45 to 90 minutes (Fig. 4E). The ability of NaCl to induce JNK phosphorylation was attenuated in cells pretreated with the 29-mer, confirming the important role of JNK signaling for the induction of MMP-9 production by NaCl.

### The 29-mer Suppresses the Expression of Proinflammatory Cytokines in EDE and in Cultured RCECs

**Figure 5.**
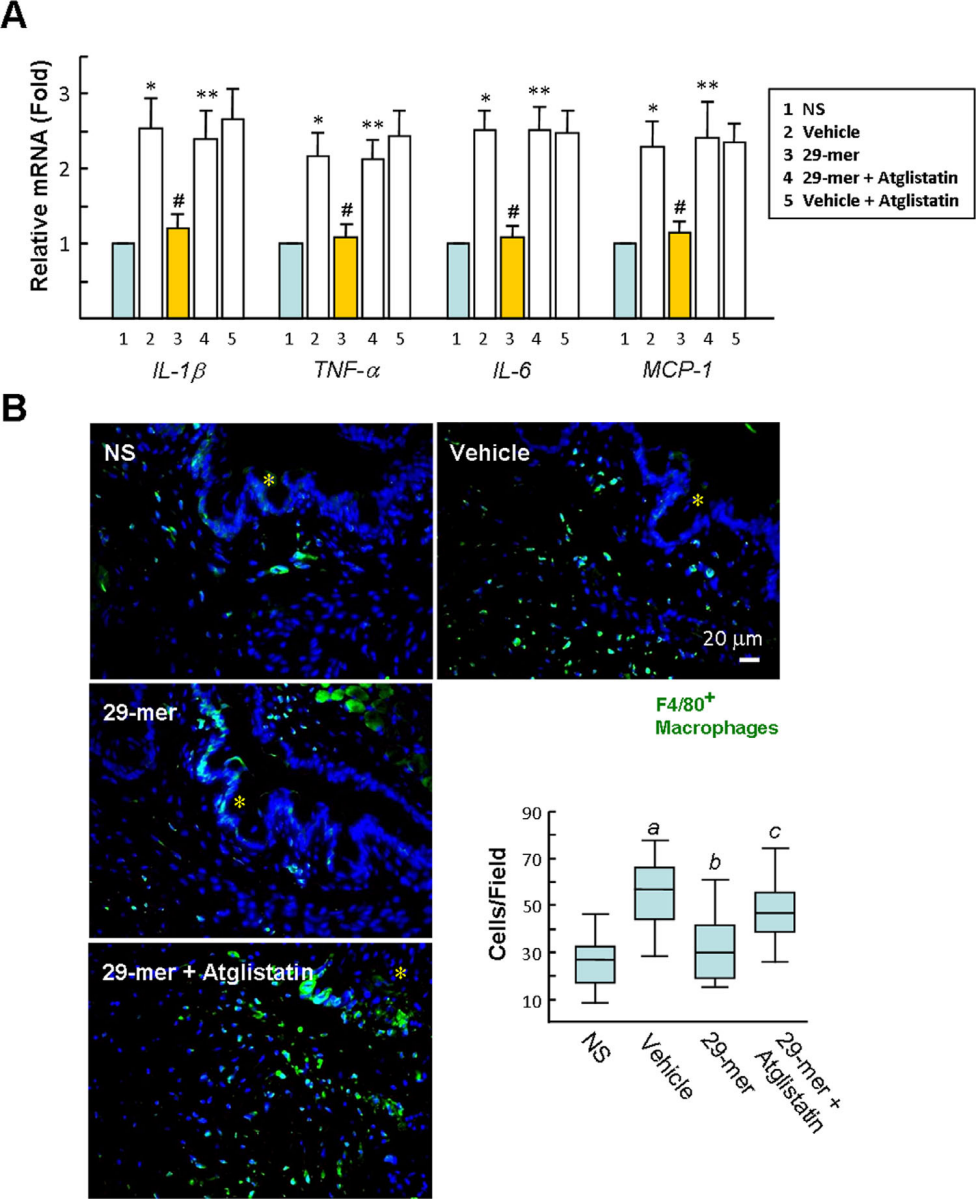
Effects of the 29-mer on the ocular surface inflammation after induction of EDE for 14 days. **(A)** qPCR analysis of the expression of proinflammatory cytokines. Data represent means ± SD of six animals in each group. *P < 0.01 versus NS group. ^#^*P* < 0.05 versus vehicle group. **P < 0.02 versus 29-mer group. **(B)** Immunofluorescence staining of F4/80 in conjunctiva and statistical analysis of numbers of F4/80-positive macrophages per field (400×) depicted by box plots. Representative pictures from two independent experiments with six mice in each group are shown. Asterisks indicate conjunctival epithelium. *^a^P =* 7.3E-10 versus NS. *^b^P* = 1.7E-6 versus vehicle. *^c^P* = 0.0002 versus 29-mer.

After EDE induction for 14 days, qPCR analysis showed that the mRNA levels of *IL-1β*, *TNF-α*, *IL-6*, and monocyte chemotactic protein-1 (*MCP-1*) in the EDE were significantly up-regulated by approximately 2.5-fold, compared with NS mice, but decreased to near baseline levels in the 29-mer group (Fig. 5A). In addition, immunofluorescence staining of F4/80 showed the numbers of macrophages in the conjunctiva were increased in EDE, compared with the NS group (Fig. 5B). This macrophage infiltration was obviously diminished in the 29-mer group. The anti-inflammatory effects of the 29-mer in EDE were reversed by atglistatin.

**Figure 6.**
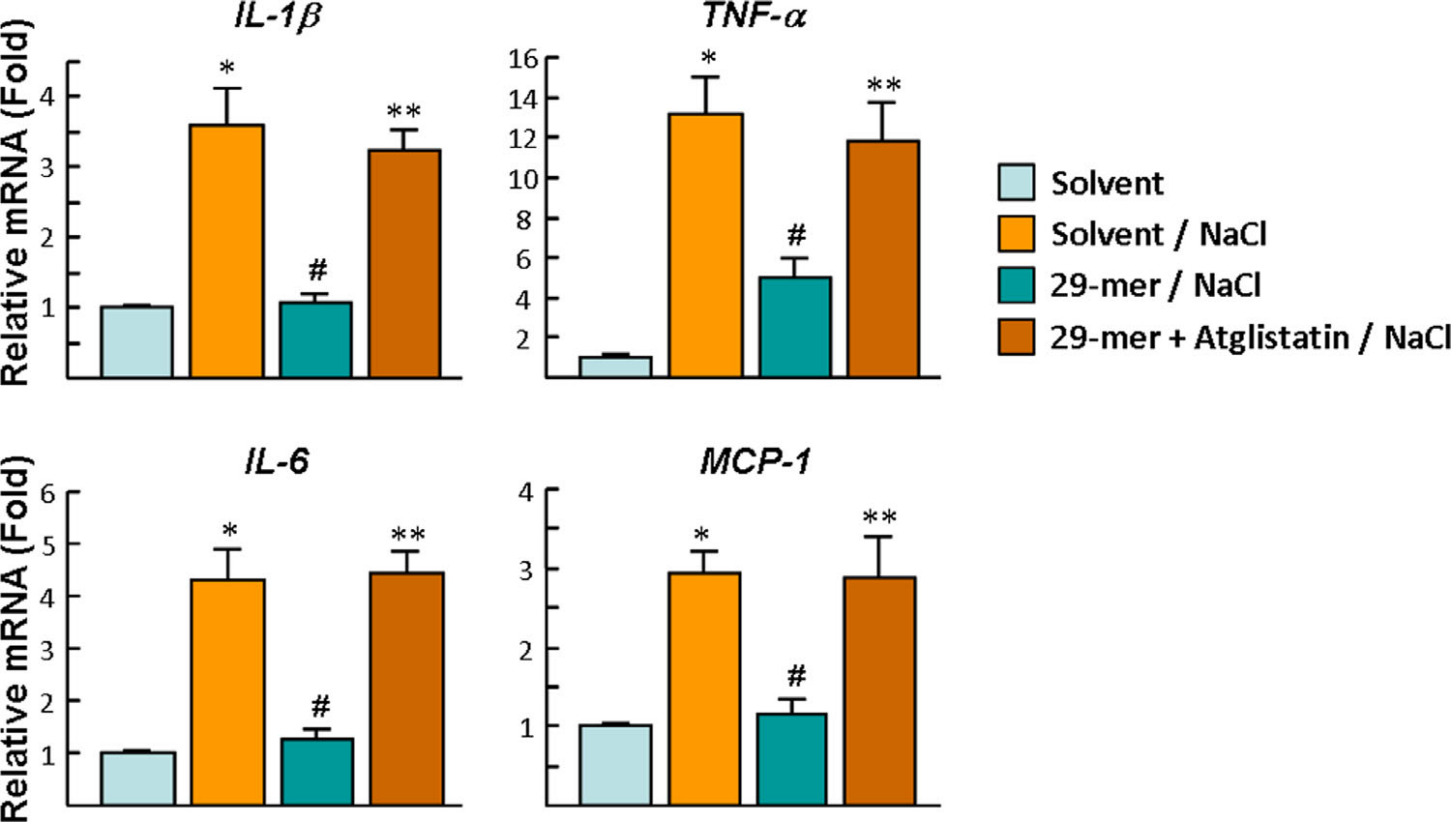
The effect of the 29-mer on the expression of proinflammatory cytokines in RCECs exposed to hyperosmotic media. Cells were pretreated with 29-mer for 6 hours and then 90 mM NaCl for a further 6 hours. Data were summarized from three separate experiments. *P < 0.001 versus solvent-treated cells. ^#^*P* < 0.0**1** versus solvent/NaCl. **P < 0.003 versus 29-mer group.

Next, qPCR analysis also showed that the gene expression of *IL-1β, TNF-α, IL-6*, and *MCP-1* in RCECs stimulated by solvent/NaCl was increased by 3.6-, 13.2-, 4.3- and 2.9-fold, compared with solvent-treatment alone, all of which were blocked by 29-mer treatment (Fig. 6). Moreover, the inhibitory effects of the 29-mer on the expression of proinflammatory genes in EDE and RCECs were reversed by atglistatin. Taken together, the results suggest that the interaction of the 29-mer with PNPLA2 suppresses the proinflammatory cytokines expressed in EDE and has a direct inhibitory effect on RCECs stimulated by hyperosmolarity.

## Discussion

Recent studies indicate that recombinant PEDF is a promising agent for the treatment of DED, via mechanisms involving immunosuppression and improvement of the stability of the tear firm lipid layer.^8^^–^^10^ Our study is the first to show that the 29-mer may constitute the core domain of PEDF responsible for its anti-inflammatory activity. Furthermore, our study shows that the 29-mer effectively suppresses MMP-9 expression in EDE and RCECs, extending our knowledge of the PEDF protective mechanisms and target cells on the ocular surface challenged by desiccation stress. In addition, the beneficial effects of the 29-mer on EDE depend on PEDF-R/PNPLA2, implying a critical role of PNPLA2 on the amelioration of inflammatory responses in the dry eye milieus stimulated by PEDF.

The increased expression of PEDF in CECs in the dry eye milieus has been suggested to be a compensatory protective mechanism to clamp down the immune responses amplified in the ocular surface.^8^ The immunosuppressive effect of PEDF leads to blockage of the maturation, migration, and antigen-presenting functions of dendritic cells, eventually leading to a reduction of several types of infiltrating immune cells in the ocular surface, such as T helper 17, CD4^+^, and CD8^+^ cells, as well as F4/80^+^ macrophages.^8^^,^^9^^,^^26^ In this regard, macrophage infiltration is important for the establishment of chronic inflammation in EDE. Local depletion of subconjunctival macrophages by injection of clodronate significantly decreases the ocular surface damage and proinflammatory cytokine expression in EDE mice.^27^^,^^28^ 29-mer treatment suppressed macrophage infiltration in EDE, further fulfilling the PEDF-mediated immunosuppressive effect.

The expression and activity of MMP-9 at the ocular surface, which is increased in patients with DED, have been shown to disrupt the tight junction proteins in the corneal epithelial barrier, effects directly associated with the conjunctival corneal fluorescein staining, sign severity values, and visual acuity scores of DED.^29^^–^^31^ An increased level of MMP-9 expression in the corneal and conjunctival epithelia has been found in EDE.^3^ Furthermore, MMP-9 knockout mice are resistant to disruption of the corneal epithelial barrier after EDE induction.^25^ Therefore, inhibition of MMP-9 to improve corneal barrier function has been suggested as a therapeutic strategy of DED.^31^ Treatment with the 29-mer considerably diminished the expression of MMP-9 in EDE, suggesting this bioactivity is an important pharmacological property of 29-mer.

It has been shown that the expression and activity of MMP-9 can be induced in primary RCECs by treatment with transforming growth factor-β and IL-1β, and be detected by gelatin zymography.^32^ Also, pharmacological inhibition of p38 MAPK or JNK signaling effectively blocks the transforming growth factor-β and IL-1β effects on MMP-9 in RCECs.^32^ In addition, JNK signaling stimulated by NaCl has been found to be critical for induction of MMP-9 in human CECs.^4^ In this study, the 29-mer was able to down-regulate JNK phosphorylation triggered by NaCl-mediated hyperosmolarity, consistent with the down-regulation of MMP-9 in RCECs. In contrast, PEDF has more than 100 potential cleavage sites, recognized by MMP-2 and -9, which result in its degradation.^33^ Given that PEDF and MMP-2 and -9 coexist in the dry eye milieus, dosing with exogenous PEDF and 29-mer to overwhelm the enzymatic activity of MMP-2 and -9 and suppress the production of MMP-9 from CECs would be beneficial for DED therapy.

Our in vitro study revealed that the inhibitory effect of the 29-mer on MMP-9 expression was abolished by atglistatin and PNPLA2 neutralizing antibody. This observation was supported by our animal study. We suggest that PNPLA2 signaling is associated with the down-regulation of MMP-9 expression. Of note, PEDF suppresses MMP-9 gene expression by MCF-7 cells, and this is reportedly blocked by a neutralizing antibody against the laminin receptor.^34^ It is likely that PEDF inhibits MMP-9 expression in various types of cells via distinct receptors. The interdependent interaction of PNPLA2 and LR and the core domain selected by PEDF are largely unclear.

In this study, we demonstrated that the 29-mer has an anti-inflammatory activity, suppressing the expression of *TNF-α*, *IL-1β*, *IL-6*, and *MCP-1* in NaCl-treated RCECs, all of which were reversed by atglistatin treatment. This finding suggests that the activation of PNPLA2 signaling by PEDF/29-mer is critical for countering the hyperosmolarity-induced responses. Similarly, PNPLA2 stimulated by PEDF has been found to block the activation of the NLRP3 inflammasome and excessive production of IL-1β in hypoxic cardiomyocytes.^35^ Excessive accumulation of proinflammatory cytokines in the ocular surface has been shown to be involved in the pathogenesis of EDE. For example, agents blocking TNF-α, IL-1, and MCP-1 receptor and CCR2 are able to suppress the development of EDE.^36^^–^^38^ In addition, conjunctival cells cultured in hyperosmolar conditions have been found to induce the expression of IL-6 that is involved in the apoptosis of conjunctival cells.^39^ These findings imply that the suppression of the proinflammatory cytokine expression in EDE is an important mechanism of 29-mer therapy for EDE.

## Conclusions

This study shows that topical application of the 29-mer has a suppressive effect on the development of EDE by inhibiting MMP-9 and proinflammatory cytokine expression. We suggest that PEDF-R/PNPLA2 is involved in this inhibition. We provide evidence that the 29-mer region is a core domain modulating PEDF anti-inflammatory signaling in the dry eye milieus and has the potential for development as ophthalmic drops to treat DED, especially in the protection of corneal epithelial integrity.
